# SARS-CoV-2’s Variants of Concern: A Brief Characterization

**DOI:** 10.3389/fimmu.2022.834098

**Published:** 2022-07-26

**Authors:** Aline Miranda Scovino, Elizabeth Chen Dahab, Gustavo Fioravanti Vieira, Leonardo Freire-de-Lima, Celio Geraldo Freire-de-Lima, Alexandre Morrot

**Affiliations:** ^1^ Instituto de Microbiologia Paulo de Goes, Universidade federal do Rio de Janeiro, Rio de Janeiro, Brazil; ^2^ Instituto Oswaldo Cruz, FIOCRUZ, Rio de Janeiro, Brazil; ^3^ Universidade La Salle Canoas, Rio Grande de Sul, Brazil; ^4^ Instituto de Biofísica Carlos Chagas Filho, Universidade Federal do Rio de Janeiro, Rio de Janeiro, Brazil; ^5^ Faculdade de Medicina, Universidade Federal do Rio de Janeiro, Rio de Janeiro, Brazil

**Keywords:** COVID-19, SARS-CoV-2, COVID-19 vaccination, viral immune escape, SARS-COV-2 variants

## Abstract

The severe acute respiratory syndrome coronavirus 2 (SARS-CoV-2) disclose the variants of concern (VOC) including Alpha (B.1.1.7), Beta (B.1.351), Gamma (P1), Delta (B.1.617.2), and Omicron (B.1.1.529). Its spike protein (S) present on the surface of the virus is recognized by the host cell receptor, the angiotensin-2 converting enzyme (ACE2) which promotes their entry into the cell. The mutations presented by VOCs are found in RBD and the N-terminal region of S protein. Therefore, mutations occurring in RBD can modify the biological and immunogenic characteristics of the virus, such as modifying the spike affinity for ACE2, increasing the virus transmissibility, or conferring the ability to escape the immune responses. The raise of a potential new SARS-CoV-2 variant capable of evading the host defenses at the same time maintaining its fitness justifies the importance of continued genetic monitoring of the pandemic coronavirus.

## Introduction

According to the World Health Organization (WHO), in December 31, 2019, the first notification of patients in Wuhan Province, China, presenting an “unknown viral pneumonia” was carried out. In January 9, 2020, the virus was isolated and its genetic sequence was shown to be a “new coronavirus”; two days later, the first death was reported by China. On February 11, 2020, the disease caused by the new coronavirus is officially named COVID-19 (Coronavirus disease 2019) (1).

The clinical symptoms of COVID-19 are fever, fatigue, cough, cardiac injury, breathlessness, sore throat, multi-organ failure, and others manifestations. The worsening of disease is associated with individual risk factors such as age, gender, cardiovascular disease, obesity and other comorbidities (2). Reports from China at the onset of outbreak and from other countries thereafter clearly demonstrated that the majority of patients (81%) have mild symptoms without pneumonia or mild pneumonia. Among patients with more significant symptoms, 14% have severe respiratory distress, and 5% respiratory failure, septic shock and/or multiple organ failure (1, 3).

COVID-19 is caused by the severe acute respiratory syndrome coronavirus 2 (SARS-CoV-2), a betacoronavirus members of the family *Coronaviridae*. SARS-CoV-2 has been characterized as a positive‐sense single‐stranded RNA. Its spike protein present on the surface of the virus is responsible for its invasion of host cells, being recognized by the angiotensin-2 converting enzyme (ACE2) in host cells which promotes their cellular entry. The ACE2 receptor is found in several mammalian organs, including lungs, heart, gastrointestinal tract, and kidney. However, the viral entry into the host cell is facilitated *via* proteolytic cleavage of ACE2 mediated by the transmembrane serine protease-2 (TMPRSS2), transforming spike protein into S1 and S2 fragments. The S1 fragment contains the receptor binding domain (RBD), a region of the protein S that binds to ACE2 (4, 5).

The rate at which mutations occur in the SARS-CoV-2 is about 10^–4^ nucleotide substitutions per site per year (6), much less than the rates for influenza virus (7) and HIV (8). However, we have observed a higher mutation rate for SARS-CoV-2 than expected. This is because the transmission rates of this virus are high, infecting a very high number of individuals in the pandemic (9). The more people infected, the greater is the likelihood of mutations appearing. These mutations may then generate viral variants, which may modify the biology of the virus, and impact of disease.

Since the beginning of the pandemic some variants has appeared and continue occurring. The World Health Organization in collaboration with partners, expert networks, national authorities, institutions and researchers have been monitoring and assessing the evolution of SARS-CoV-2 since January 2020. This informal group named Technical Advisory Group on Virus Evolution (TAG-VE), supports a larger WHO-coordinated global risk monitoring and assessment framework for SARS-CoV-2 variants, which include other WHO advisory groups, such as the Expert Advisory Group on COVID-19 Vaccine Composition (TAG-CO-VAC), the Strategic Advisory Group of Experts on Immunization (SAGE), and the Strategic and Technical Advisory Group for Infectious Hazards (STAG-IH) (9).

The TAG-VE shall have up to 30 members, these have experience in virology, bioinformatics, epidemiology, laboratory sciences, pharmacology, clinical management, or in one or several diagnostic, therapeutic and/or vaccine products. In the selection of the TAG-VE Members, consideration shall be given to attaining an adequate distribution of technical expertise, geographical representation and gender balance (9). The group periodically monitors the appearance of new mutations and their impacts on the biology of the virus, evaluating the emergence of variants, their transmissibility, disease severity, diagnosis and treatment, in an attempt to contain the spread of these new potential variants. According to their clinical impact on the pandemic, they are classified as either interest or concern variants (10, 11).

Variants of interest are defined as those with genetic changes that are predicted or known to affect virus characteristics such as transmissibility, disease severity, host immune escape, diagnostic or therapeutic resistance. They are identified to cause significant community transmission or multiple COVID-19 clusters, in multiple countries with an increasing relative prevalence alongside the number of new cases over time, or other apparent epidemiological impacts to suggest an emerging risk for global public health, as it happened for the Lambda variant (10).

The variants of concern, besides having all the characteristics of the variants of interest, can show a sort of different characteristics including increased rates in transmissibility or detrimental change in COVID-19 epidemiology; increased virulence or change in clinical disease symptoms; decrease in the effectiveness of public health and social measures or available diagnostics, vaccines, therapeutics. The major viral mutants identified to date exhibiting one or more of these characteristics (**Table 1**) are named Alpha (B.1.1.7), Beta (B.1.351), Gamma (P1), Delta (B.1.617.2) and Omicron (B.1.1.529) variants (10).

**Table 1:** Variants and spike mutations, including specific RBD mutations. The most important RBD positions that interacts with ACE2 are E484, S494, N501, K417, L452 (12–14).

Variants	Deletions	Mutation S1+S2	RBD mutations	Earliest Documented in
Alpha (B.1.1.7)	ΔH69, ΔV70, ΔY144	N501Y, A570D, D614G, E484K, T716I, S982A, P681H, D1118H	E484K, S494P, N501Y	United Kingdon (10, 15)
Beta (B.1.351)	Δ241/4, Δ242, Δ243	N501Y, A701V, D614G, E84K, D215G, K417N, and D80A	K417N, E484K, N501Y	South Africa (10, 16)
Gamma (P1)		N501Y, L18F, D614G, E484K, T1027I, K417T, D138Y, R190S, H655Y, P26S, T20N	K417N, E484K, N501Y	Brazil (10, 17, 18)
Delta (B.1.617.2)	Δ156, Δ157	T478K, L452R, D614G, G142D, D950N, T19R, P681R, R158G, E484Q	L452R, E484Q	India (10, 19)
Omicron (B.1.1.529)	ΔH69-V70, Δ143-145, Δ211-212	A67V, T95I, G142D, G339D, S371L, S373P, S375F, K417N, N440K, G446S, S477N, T478K, E484A, Q493K, G496S, Q498R, N501Y, Y505H, T547K, D614G, H655Y, N679K, P681H, N764K, D796Y, N856K, Q954H, N969K, L981F	K417N, E484A, N501Y	South Africa (10, 20)

Variants and spike mutations, inlcuding specific RBD mutations. The most important RBD positions that interacts with ACE2 are E484, S494, N501, K417, L452 and the countries each of the variants have been initially reported.

## Alpha Variant

**Figure 1:**
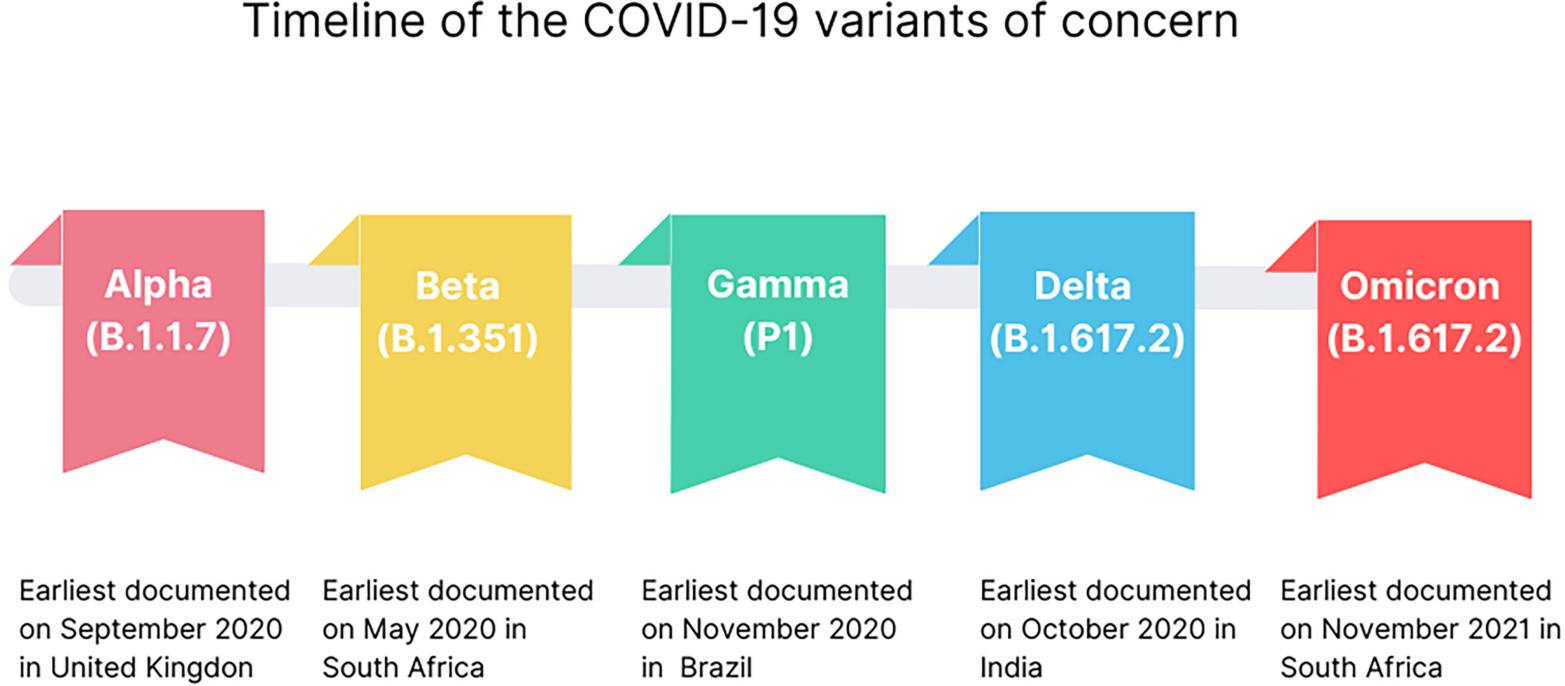
Time line emergence of COVID-19 VOCs.

**Figure 2:**
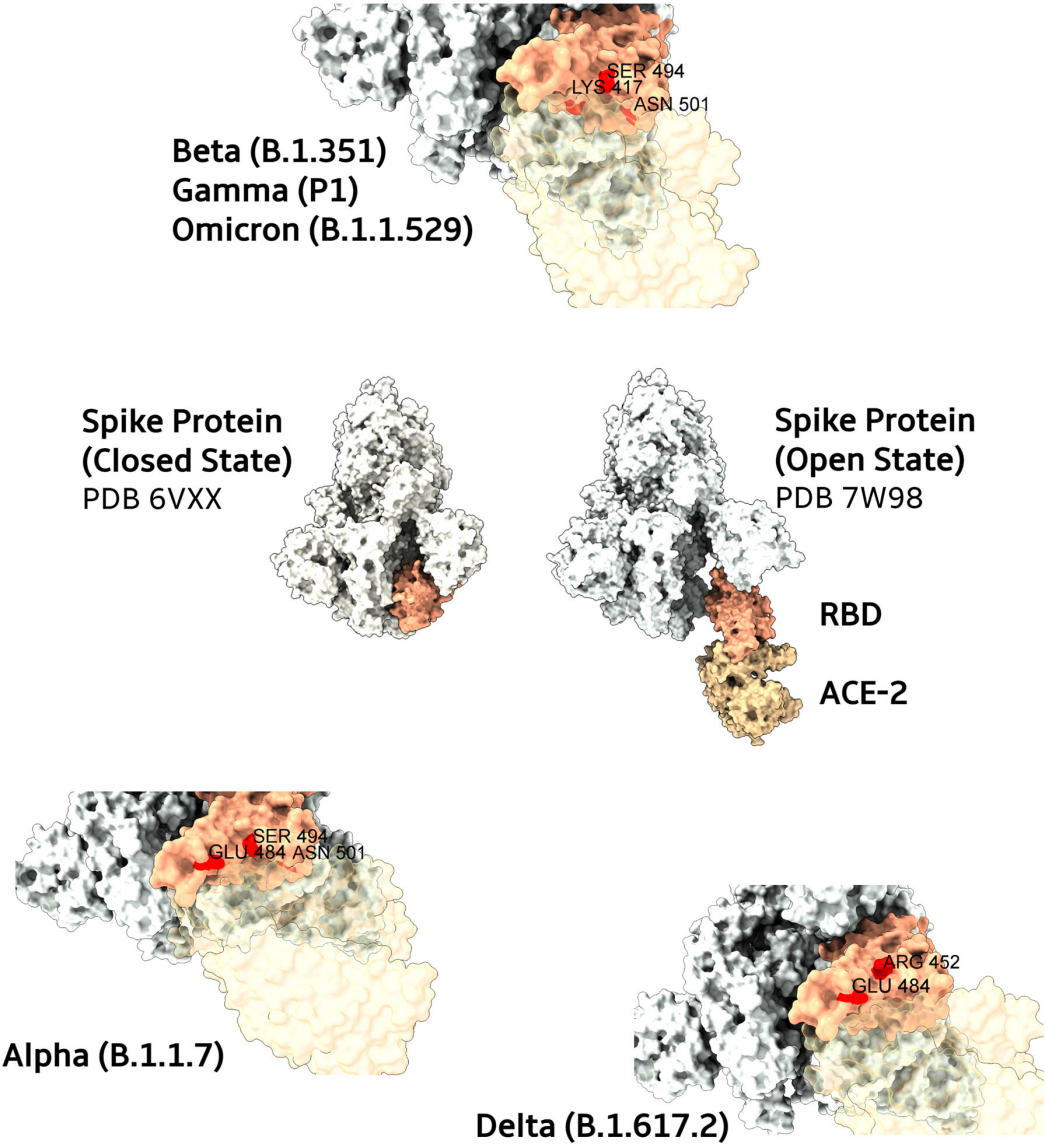
Main mutations present in SARS-CoV-2 Spike protein variants. In the center, structures from Spike proteins (colored in ivory) in their closed and open states (complexed with ACE2, in light brown). The RBD domain is highlighted in salmon. Closes from the ACE2 interaction interfaces of RBD domains are depicted for Beta, Gamma, and Omicron (top), Alpha (bottom left), and Delta (bottom right) variants. Mutated residues are colored in red with their respective name and positions. To improve the visualization of variants mutated regions, transparency was applied to ACE2 protein surfaces.

The Alpha variant (B.1.1.7) was dominant in the United Kingdom in early 2021 (**Figure 1**), containing several deletions and mutations in its spike protein (**Figure 2**) such as N501Y, A570D, ΔH69/ΔV70, ΔY144, P681H, T716I, S982A, and D1118H. The N501Y substitution, common to the other variants, seems to be associated with greater transmissibility, as it has a greater affinity for ACE2. After the first identification of B1.1.7 phylogeny, an E484K mutation in the protein S was identified (15). This replacement, as well as the N501Y appears to increase the spike affinity to ACE2, being related to resistance to antibody neutralization targeting the original epitope (21). N501Y mutation slowed the dissociation of the RBD from the ACE2 receptor, resulting in a fourfold greater affinity than wild-type RBD (22).

*In vitro* studies with serum from patients vaccinated with BNT162b2 or ChAdOx1 has demonstrated that mutation in the spike protein reduce the antibody neutralization of B.1.1.7 variant as compared to the original virus (23, 24). However, other studies show little or no difference in the neutralization antibody capacity of the plasma of vaccinated individuals against the B.1.1.7 variant compared to the original strains (25–27).

In Qatar, the BNT162b2 vaccine effectiveness was estimated with a test-negative case–control study design. This study showed that the effectiveness of this vaccine against the alpha variant was 89.5% to develop the disease and 97.4% against severe and fatal cases of the disease (28).

In Brazil, the effectiveness of ChAdOx1 and CoronaVac/Sinovac was evaluated in two outbreaks of the alpha variant, in a convent and a Long Term Care Facility for the elderly. They observed that vaccination did not prevent people from becoming infected, but there was a reduction in the number of deaths, symptoms and severe cases of the disease, even in this population whose average age was around 77 years, with 1 dose of ChAdOx1 or two from CoronaVac/Sinovac (29).

In UK, a group evaluated a cohort that had already participated in the ChAdOx1 vaccine efficacy trials. These participants received booster doses, according to the vaccination protocol, and periodically provided upper airway swabs on a weekly basis and also if they developed symptoms of COVID-19 disease. In this work, they showed that although the neutralization assays with the plasma of these individuals showed a reduction in neutralization against this variant, the clinical data show an efficacy of the ChAdOx1 vaccine of approximately 70% against the symptomatic cases. This indicates that either a low neutralization rate is already sufficient for protection, there may be complement activation and elimination of infected cells, antibody-dependent NK cell activation, macrophage opsonization, or other immunological mechanisms such as induction of T cells specific for spike (30).

## Beta Variant

The Beta variant (B.1.351) was identified in South Africa, appearing after the first wave of the epidemic, becoming prevalent in three provinces (the Eastern Cape, Western Cape and KwaZulu–Natal), during the second wave (**Figure 1**). The Beta variant has eight mutations in the spike (**Figure 2**), three of which are known and common to other variants, such as K417N, E484K and N501Y substitutions, affecting key sites of RBD associated with escape of neutralizing antibodies (31). It is not known whether this combination confers a greater affinity to the host invasion ACE2 receptors. In addition to RBD, the N-terminal domain (NTD) of spike is a target site for antibody neutralization, as mutations in this region show substantial or complete escape from neutralizing antibodies (16).

Recently, a group using a live-virus neutralization assay has compared the neutralization of a non-variant of concern to Beta variant, using plasma collected from adults who were hospitalized with COVID-19 during the two waves of infection in South Africa. The results showed that the Beta variant was efficiently neutralized by plasma from infected individuals in the second wave, but there was a decrease, around 15 times, in the neutralization efficiency when plasma collected from infected individuals who was diagnosed in the first COVID-19 wave (32).

Studies on the effectiveness of available vaccines against the strains of concern found the Oxford-AstraZeneca vaccine with only 10% protection against mild-to-moderate disease associated to Beta variant in young populations with an average median age about 30 in South Africa (33). In contrast, Johnson & Johnson’s vaccine showed 64% protection against moderate-to-severe clinical forms of disease (34). The Pfizer/BioNTech was reported to be less effective against Beta variant than other variants based on a small analysis of breakthrough infections in Israel (35). However, a test-negative case–control study design carried out in Qatar showed that the effectiveness of this vaccine against the beta variant was 75%, and against severe and fatal cases, the effectiveness was 97.4% (28).

## Delta Variant

The Delta variant, Lineage B.1.617, was first identified in India in the Maharasha city (**Figure 1**), and was shown to have the highest transmissibility, when compared to the Alpha (B.1.1.7), Beta (B.1.351), and Gamma (P.1) variants. People infected with this variant have a viral load about 1,000 times greater than people infected with the wild strain virus (SARS-CoV-2 WT), in addition to its higher replicating rate. This explains why this variant is so transmissible, and why it became the dominant variant worldwide, until the emergence of the Omicron variant (19).

The Delta variant belongs to the B.1 strain, which contains a mutation in the spike protein (D614G) that already exists in the alpha and beta variants, indicating that it can increase the affinity of the Spike protein for the host ACE2 receptors (36, 37). The B.1.617 contains 3 sub lineages identified as B.1.617, B.1.617.2, and B.1.617.3, with B.1.617.2 being the most transmissible in humans. The B.1.617 has also many other mutations inside the lineage. In the spike protein (**Figure 2**), mutations are found in RBD (L452R and T478K), NTD (R158G, T19R, G142D, Δ156-157), S2 region (D950N), and a mutation at the site close to furin cleavage. These mutations may increase the efficiency of replication as well as the regulation of S protein, thus reducing the chances of being recognized by neutralizing antibodies (36).

Recent studies has tested the efficacy of Pfizer/BioNTech and Oxford-AstraZeneca vaccines to Delta variants, showing reduced sensitivity of SARS-CoV-2 variant Delta to antibody neutralization (36, 38). However, a test-negative case–control study design carried out in Israel showed that the effectiveness of these vaccines after the complete vaccine program was similar to the efficacy of clinical trials (39).

In China, due to a public policy of zero tolerance, in 2020 practically all cases of COVID-19 were imported, with almost no internal transmission of the virus. In this way, the immunity of the Chinese population is practically all induced by the vaccine formulations used in the country, which allows a good assessment of the effectiveness of vaccines, in the isolated outbreaks that occurred in 2021 as a result of cases of the Delta variant. This work evaluated the efficacy of two attenuated virus vaccines, CoronaVac/Sinovac and BBIBP-CorV vaccine, as well as an adenovirus vector vaccine, Can-sino’s Ad5 vaccine, against this variant in this 2021 outbreak, which occurred near Yunnan province. They used a retrospective cohort design among close contacts of infected individuals to determine vaccine effectiveness. They observed that the two inactivated virus vaccines were 74.6% effective against symptomatic COVID-19, and 100% against severe cases of the disease. The same was observed for the adenovirus vector vaccine (40).

A meta-analysis study corroborates these results. This article brought together five papers on vaccine effectiveness from Pfizer/BioNTech, three from Oxford-AstraZeneca and one from CoronaVac/Sinovac. Overall, the three vaccines were effective after the two doses, the Pfizer/BioNTech had approximately 83% of effectiveness after the second dose, and 97% after the third dose. For Oxford-AstraZeneca the effectiveness was approximately 80%, and for CoronaVac/Sinovac approximately 65% after the second dose, and 63% after the third dose. For severe forms of the disease, vaccine effectiveness was approximately 98% for Pfizer/BioNTech, 91% for Oxford-AstraZeneca and 75% for CoronaVac/Sinovac (41).

## Gamma Variant

The Gamma variant was detected in Japan in travelers arriving from Brazil in January 2021 (17) (**Figure 1**). By April it had already been worldwide spread in 36 countries, with local transmission occurring in five, including Brazil. This variant differs from the original Wuhan strain presenting twelve mutations in the spike (**Figure 2**), including N501Y, K417T and E484K (18). Despite having an accumulation of three different mutations, it seems that this variant has replication potential similar to the original strain, and is likewise neutralized with serum from individuals vaccinated with the Pfizer/BioNTec vaccine (18).

The Gamma strain has also been linked to reinfection cases in Manaus, Brazil, suggesting its efficiency to circumvent the acquired immune response from other previous strain infections. This may explain the rapid spread of the Gamma variant in Brazil. In Manaus, at the end of 2020, we have observed a drastic increase in the number of cases for this variant, and consequently in the number of deaths during pandemic. Analyzes integrating viral genomic tracking and mortality data estimate that Gamma variant it is about 1.7 to 2.4 times more transmissible as compared to previous SARS-CoV-2 non-Gamma infection.

One study evaluated the potential of neutralizing antibodies after two doses of CoronaVac/Sinovac vaccine against the original B.1 strain and the Gamma variant. Antibody titers in individuals who had received two doses of CoronaVac/Sinovac 21 days before sample collection were near 1:80 for B.1 and 1:20 for P.1 isolates. Collectively, the data suggest that P.1 lineage virus might escape from neutralizing antibodies induced by an inactivated SARS-CoV-2 vaccine, especially at 5 months after vaccination as immunity wanes (42).

Previous non-P1 lineage infections can induce about 54-79% protection against P1 variant (43). The efficacy of the CoronaVac/Sinovac inactivated virus vaccine in Brazil, where 75% of infections has occurred in the presence of Gamma variant, was estimated about 50% protection against symptomatic infection (44).

A test-negative case–control study design evaluated the efficacy of CoronaVac/Sinovac in elderly people over 70 years of age during the Gamma variant outbreak in São Paulo, Brazil. The data show that this vaccine is 47% effective against symptomatic COVID-19, 57% against severe COVID-19 and 67% against deaths (44).

A multicentric study carried out in Brazil to assess the effectiveness of the Oxford-AstraZeneca vaccine in preventing symptomatic COVID-19 showed that against the Gamma variant this vaccine was 64% effective, but the number of confirmed cases of this variant was small, therefore this estimate is weak (45).

## Omicron Variant

The new Omicron variant (B.1.1.529) was first detected in Botswana, on November 11, 2021(**Figure 1**). Soon after, it was detected in Hong Kong in a patient who had arrived from a trip to South Africa (10). In these countries, cases had highly increased from one week to another after its detection, which was partially attributed to the increase in surveillance (20). The Omicron has some deletions, such as 69–70del, which prevents Spike from being detected by the RT-PCR test. It also shows more than 30 mutations in the S protein (**Figure 2**), with about 15 ones present in the RDB, some of them in regions that overlap the mutations of the other variants of concern, such as those that occur in residues K417, E484, and N501 (10). These deletions and mutations are associated with increased transmissibility by increasing the affinity of the spike protein with ACE2, and also associated to host immune escape and reduced neutralization of vaccine-induced antibodies (46–49).

Studies have indicated that the third dose of Pfizer/BioNTech and Moderna mRNA vaccines are efficient to neutralize the Omicron variant. The first and second doses of these vaccines resulted in low to none neutralization for this variant. However, patients who received a third dose exhibited efficient neutralization against the variant (50). The same was observed when the booster was heterologous, with the vaccination schedule being CoronaVac/Sinovac and the booster (third dose) of Pfizer/BioNTech (51), or the vaccination schedule of Oxford-AstraZeneca and the booster of Pfizer/BioNTech (52).

Furthermore, *in vitro* infection experiments demonstrated that the Omicron pseudovirus also depends on the human ACE2 receptor for target cell entry and infects host cells four times more efficiently than the wild-type pseudovirus, and 2 times more when compared to the Delta pseudovirus (50). Corroborating these findings, other studies showed that Omicron had a growth advantage over Delta in Gauteng, South Africa, where it presented 5.4-fold weekly increase in clinical cases as compared to Delta variants (49).

In general, infection with the Omicron variant appears to cause milder symptoms in relation to other variants. In countries where the vaccination programs are advanced it can be demonstrated high index of vaccine protection in all analyzed populations. However, in countries where vaccination is still incipient, the spreading of Omicron variant is associated with an increased number of infection cases, but this epidemiology is not accompanied by an increase in mortality rates, still indicating a low virulence of this variant. In fact, the replication capacity of Omicron is significantly attenuated *in vitro* and *in vivo* as compared to others such as SARS-CoV-2 WT, Alpha, Beta and Delta variants. This is explained by its lower efficiency in using TMPRSS2, as mutations in the spike gene of Omicron variant cause its inefficient cleavage by the host protease, leading to reduced recognition by host protease (53).

## Implications on Both Host Immune Response and Vaccine Efficacy

Some mutations capable of altering the fitness of SARS-CoV-2 were detected in the Spike protein early in the pandemic, due to an international effort in viral monitoring worldwide (10). Some of them are located in the RBD (**Figure 2**). Spike protein is the main neutralizing target for antibodies generated after SARS-CoV-2 infection (more than 90% of neutralizing antibodies) (54), especially the RBD and is the SARS-CoV-2 component of mRNA and adenovirus-based vaccines licensed for use (55–57). The immunodominant epitopes are present in the region of the RBD that overlaps the ACE2 binding site (54).

The interaction of the Spike protein with the host cell occurs dynamically in a three-dimensional structure. Neutralizing antibodies can act in different ways, first by blocking the binding site (RBD) with ACE2 (58, 59). ^A^ few of these, bind to a motif surrounding the N-linked glycan at residue 343. These antibodies, exemplified by S309 (60), do not block ACE2 interaction, and destabilizing the S-trimer may be their mechanism of action. Neutralizing anti-NTD mAbs do not block ACE2 interaction and bind to a so-called supersite on the NTD (61, 62), however, they generally fail to provide a broad protection as the supersite is disrupted by a variety of NTD mutations present in the variants of concern (VOC).

The main vaccines applied today aim to induce an immune response, either humoral or cellular, against the spike protein. This is the case of Oxford-AstraZeneca (55), Pfizer/BioNTech (56) and Moderna (57). The variants of concern have mutations in RBD and the N-terminal region of S protein. Therefore, mutations occurring in RBD could potentially modify the biological and immunogenic characteristics of the virus, may affecting the spike affinity for ACE2, thus affecting the virus transmissibility, or conferring the ability to escape the immune response (63). This may affect the effectiveness of these vaccines. These mutations in the VOCs, without changing the viral fitness, can be selected during the course of the infection, by the host’s immune system, by therapy with convalescent plasma, by vaccines and also by treatment with antibodies (64).

Vaccination induces a humoral and cellular response, so it is plausible to assume that the production of antibodies against Spike, preventing its binding to ACE2 and consequently infection, is an important mechanism in the control of the disease (65). Several studies show that vaccines developed against COVID-19 are capable of inducing neutralizing antibodies against Spike. It is not known, however, the amount of neutralizing antibodies needed to control the infection (66), there seems to be a relationship of the higher the neutralizing antibody titers, the greater the protection (67).

Since neutralization is an important mechanism for infection control, concern about variants is legitimate. Some works show that there is a reduction in neutralization by antibodies in *in vitro* assays for existing variants. However, in clinical practice, vaccines are being effective in reducing severe cases of the disease. This only reinforces the doubt about the necessary amount of neutralization to control the disease, as well as its relevance, since vaccines also induce a specific T response to the spike protein (68).

## Concluding Remarks

Mutations would have evolutionary relevance if they could promote phenotypic changes in the viral behavior that promote its susceptibility to natural selection. In most RNA viruses, the variations that confer greater ability to evade the immune system are usually associated with increased fitness, that is, their ability to infect the host and being transmitted. This could be the case of antigenic changes causing viral evasion responses to host defense thus subverting the neutralizing action of antibodies, induced by a natural immune or vaccine responses (69). The HIV virus, for example, is one of the viruses with the greatest capacity to produce high fitness-variants capable of evading the host immune system.

However, when the selective pressure occurs under reverse transcriptase or protease, due to treatment with antiretroviral drugs, the variants that appear resistant to these drugs have a reduced replicative capacity and transmission (70). Influenza`s virus hemagglutinin, for instance, is extremely immunogenic and any variation on it allows evasion of the immune system, without loss of fitness (71). Therefore, much attention is currently given to the continued genetic monitoring of new SARS-CoV-2 variants of concern that could be able to evade the host immune defense mechanisms and promote an deadly wave of epidemiological outbreak (69).

## Author Contributions

AM, ECD, GFV, LFL, CGFL and ED wrote the manuscript. All authors contributed to the article and approved the submitted version.

## Conflict of Interest

The authors declare that the research was conducted in the absence of any commercial or financial relationships that could be construed as a potential conflict of interest.

## Publisher’s Note

All claims expressed in this article are solely those of the authors and do not necessarily represent those of their affiliated organizations, or those of the publisher, the editors and the reviewers. Any product that may be evaluated in this article, or claim that may be made by its manufacturer, is not guaranteed or endorsed by the publisher.
